# A novel phenoxy thiophene sulphonamide molecule protects against glutamate evoked oxidative injury in a neuronal cell model

**DOI:** 10.1186/1471-2202-14-93

**Published:** 2013-09-02

**Authors:** Nailya S Gliyazova, Eun Y Huh, Gordon C Ibeanu

**Affiliations:** 1 BRITE, North Carolina Central University, 1801 Fayetteville Street, Durham, NC 27707, USA; 2 Center for Gastrointestinal Biology and Diseases, University of North Carolina, Chapel Hill, NC 27599, USA; 3 Department of Pharmaceutical Sciences, North Carolina Central University, 1801 Fayetteville Street, Durham, NC 27707, USA

**Keywords:** Glutamate, Neuroprotection, Excitotoxicity, Small molecule, Alzheimer’s disease, Oxidative stress, ERK3, Neurodegenerative disease, Phenoxy thiophene, HT-22

## Abstract

**Background:**

Glutamate is one of the major neurotransmitters in the central nervous system. It is a potent neurotoxin capable of neuronal destruction through numerous signal pathways when present in high concentration. Glutamate-evoked excitotoxicity has been implicated in the etiology of many neurodegenerative diseases including Alzheimer’s disease (AD), Parkinson’s disease (PD), and ischemic stroke. Increasing evidence has shown that reactive oxygen species (ROS) provoked by glutamate-linked oxidative stress plays a crucial role in the pathogenesis of these disorders. We previously reported the discovery of an aryl thiophene compound, 4-chloro-N-(naphthalen-1-ylmethyl)-5-(3-(piperazin-1-yl)phenoxy)thiophene-2-sulfonamide (B355252) from a proprietary library of small molecules. We showed that this compound was capable of potentiating nerve growth factor (NGF)-primed neurite outgrowth in neuronal cell models in a low NGF environment. In the present study we investigated the neuroprotective effects and signaling pathways of B355252 on glutamate-evoked excitotoxicity in HT-22, a murine hippocampal neuronal cell line.

**Results:**

Glutamate significantly decreased HT-22 neuronal cell viability in a concentration-dependent manner as measured by the MTT assay. Co-treatment with 2, 4, and 8 μM B355252 protected against cell death caused by glutamate-induced toxicity by 9.1% (p<0.01), 26.0% (p<0.001), and 61.9% (p<0.001) respectively, compared to glutamate-treated control group. B355252 at a concentration of 8 μM fully rescued HT-22 from the neurototoxic effects of glutamate, and by itself increased cell viability by 16% (p<0.001) above untreated control. Glutamate enhanced reduction in glutathione (GSH) synthesis was reversed by 15% (p<0.01) in the presence of B355252. B355252 reduced the expression of apoptosis inducing factor (AIF) by 27%, while the proapoptotic Bcl-2 associated X protein (Bax) was strongly attenuated 3-fold. Glutamate-evoked increase in intracellular calcium (Ca^2+^) load and subsequent ROS production was inhibited by 71% (p<0.001) and 40% (p<0.001) respectively, to comparable level as untreated control in the presence of B355252. Glutamate significantly upregulated the phosphorylation of extracellular signal regulated kinase Erk1/2 (pERK1/2), while decreasing Erk3. In contrast, B355252 potently attenuated the glutamate-dependent activation of Erk1/2 and robustly increased the level of ERK3 in HT-22.

**Conclusions:**

A novel phenoxy thiophene small molecule, B355252, suppresses glutamate-evoked oxidative stress in HT-22 neurons by blocking Ca^2+^ and ROS production, and altering the expression or phosphorylation states of Erk kinases. This molecule previously reported to enhance neurite outgrowth in the presence of sub-physiological concentrations of NGF appears to be a promising drug candidate for development as a potential therapeutic and neuroprotective agent for various neurodegenerative disorders.

## Background

Several neuropathological processes are associated with glutamate excitotoxicity and oxidative stress that lead to neuronal damage and death. Despite concerted effort in recent years to develop new and effective drugs to combat diseases triggered by glutamate excitotoxic cascade, there have been few therapeutic advances in the treatment of these devastating conditions. Glutamate is the most abundant neurotransmitter in the brain and plays a crucial role in neuronal tissue damage during cerebral ischemic hypoxia caused by toxic levels of the neurotransmitter in the central nervous system [1]. The resulting decrease in ATP levels under toxic glutamate conditions leads to failure of energy-dependent sodium pumps, anion channels, membrane depolarization, and glutamate secretion.

The tonic basal concentration of extracellular glutamate in the brain under normal physiologic condition has been estimated in the range 1–30 μM [2]. This concentration determines its role in metabolic processes. Contradictory reports exist in the literature on the brain levels of glutamate in neuropsychiatric disorders. Previous reports point to decreased levels of glutamate in Alzheimer’s disease [3,4], while increased plasma levels of glutamate have been reported in epilepsy, Alzheimer’s disease, and amyotrophic lateral sclerosis [5-7]. In the case of Alzheimer’s disease, recent research suggests a mechanism whereby amyloid beta decreases the uptake of glutamate at the synapse resulting in excess glutamate in the extracellular space outside the synaptic terminal. The excess glutamate leads to activation of glutamate receptors and is thought to play a role in the pathophysiology of the diseases.

Glutamate exerts its effects via three membrane proteins composed of two major classes of receptors and a cystine/glutamate antiporter protein. Under normal physiological conditions, glutamate released in the synaptic cleft binds the two major types of post-synaptic glutamate receptors, the metabotropic glutamate receptors (mGluR) and ionotropic glutamate receptors (iGluR). The iGluR consists of three members: N-methyl-D-Aspartic acid (NMDA), α-amino-3-hydroxy-5-methyl-4-isoxazolepropionic acid (AMPA), and kainic acid (KA) receptors, while the mGluR has eight members (mGluR1-8). The mGluRs in concert with the iGluRs facilitate synaptic plasticity, learning, memory and other cognitive functions [8,9].

Glutamate-induced cell death is mediated in part by accumulation and overstimulation of the postsynaptic glutamate receptor system [10]. Chronic exposure of neurons to glutamate results in persistent activation of glutamate receptors, which destabilizes the tightly controlled mechanisms that regulate Ca^2+^ homeostasis in neurons. Perturbation of intracellular Ca^2+^ balance drives the accumulation of Ca^2+^ ions in the mitochondria and leads to bioenergetic failure as a result mitochondrial membrane depolarization linked to opening of the mitochondrial permeability transition pore. The subsequent activation of Ca^2+^-dependent enzymes negatively impacts a large number of Ca^2+^ mediated functions and lead to a cascade of events that culminate in neuronal injury and cell death [11,12]. There is abundant evidence that prolonged exposure to high concentrations of extracellular glutamate promotes oxidative toxicity in cells that do not express functional iGluR such as HT-22 neurons, primarily by activation of mechanisms that negatively impact the function of the cystine/glutamate antiporter [13]. The resulting decrease in cystine uptake across the cell membrane lowers intracellular levels of the free radical scavenger glutathione (GSH). Depletion of GSH leads to oxidative stress that is accompanied by downregulation of the cystine-dependent antioxidant system, formation of ROS, and alteration in Ca^2+^ homeostatic mechanisms resulting in cell death [14].

Oxidative stress has emerged as a major mechanism that underlies the etiology of a variety of neuropathological disorders, including ischemic stroke, traumatic brain injury (TBI), depression, Alzheimer’s disease, Parkinson’s disease [15-20]. Most neurodegenerative diseases are characterized by progressive neuronal atrophy and cell death in the central and peripheral nervous system. In the case of Alzheimer’s disease, the leading cause of dementia in the elderly population, there is a significant loss of cholinergic neurons in brain regions associated with learning and memory including the hippocampus, amygdala, and cortex, resulting in decline in cognitive, behavioral, and functional abilities. Past studies suggest that oxidative damage to proteins, lipids, and DNA may contribute to neuronal loss in Alzheimer’s disease [21,22]. Oxidative stresses triggered by amyloid beta (1–42) peptide induce an increase in nitric oxide synthase, formation of ROS, and alteration of mitochondrial dynamics [23-25]. More recently, the role of amyloid beta peptide in elevating intracellular Ca^2+^ levels through mechanisms involving NMDA and AMPA receptors resulting in disregulation of synaptic transmission and cell death was demonstrated in the early phase of Alzheimer’s disease [26,27]. Taken together, these studies suggest a potential role for oxidative stress and regulation of calcium homeostasis as a possible factor contributing to the death of cholinergic neurons in Alzheimer’s disease.

Previously, we reported the identification of an aryl-thiophene compound (B355252) that potentiates NGF-primed neurite outgrowth (NOG) in NS-1 cell, a derivative of the pheochromocytoma PC12 cell line [28]. This compound is devoid of NOG properties alone but promotes the differentiation and elongation of axonal-like processes *in vitro* in the presence of sub-physiological concentrations of NGF as exists in brain regions affected by Alzheimer’s disease. In the present study, we investigated the neuroprotective effect of B355252 in an oxidative glutamate excitotoxicity model in HT-22 neuronal cell line, and sought to elucidate the underlying molecular pathway.

## Results

### Prolonged exposure of HT-22 to glutamate triggers dose-dependent cytotoxic effect

**Figure 1:**
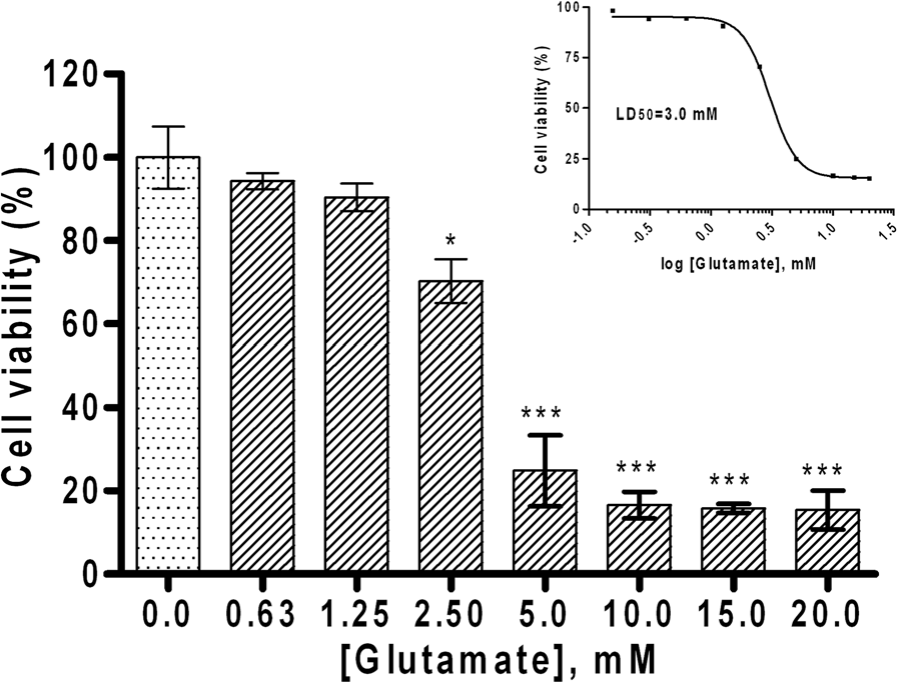
**Glutamate-dependent toxicity in HT-22 cells is concentration dependent.** HT-22 was treated with the indicated concentrations of glutamate and cell viability assessed with MTT assay. Cells exposed to glutamate concentrations greater than 2.5 mM showed significant decrease in cellular viability. Glutamate exhibited a LD_50_ of 3.0 mM in HT-22 (inset). Values represent the means ± SD as a percent (%) of control (*p<0.05, ***p<0.001).

We first determined the toxic effect of glutamate in HT-22 cultures in concentration-dependent assays. Cell viability was measured with MTT. Glutamate treatment of HT-22 led to progressive significant reduction in cell viability with increasing glutamate concentration (Figure 1). At 2.5 mM glutamate dose the number of viable cells decreased by roughly 25% (p<0.05) compared to untreated cells. When glutamate concentration was doubled to 5 mM, cell viability decreased by 75% (p<0.001) compared to the untreated cultures. At 10 mM glutamate, the viability of HT-22 decreased by nearly 83% (p<0.001) of untreated cells with no additional toxicity observed when glutamate was increased to 15 mM and 20 mM. The median lethal dose (LD_50_) of glutamate for HT-22 in this experiment is 3.0 mM (Figure 1 inset).

### Exposure of cells to B355252 prevents glutamate-induced excitotoxicity

**Figure 2:**
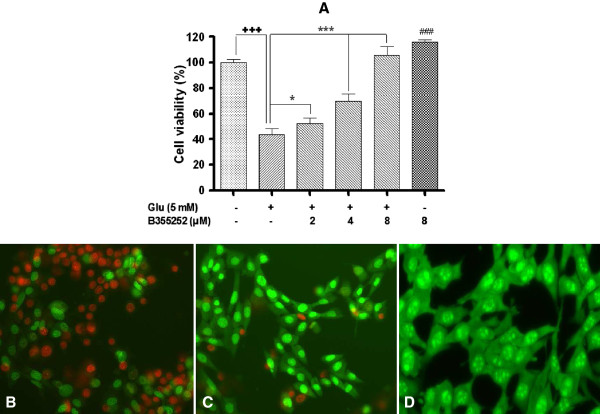
**Neuroprotective effects of B355252 against glutamate-induced toxicity.** HT-22 cells were treated with or without glutamate and the indicated concentrations of B355252 and analyzed with the MTT assay **(A)**, or a fluorescent viability assay **(B, C, D)**. **(A)**. B355252 conferred significant dose-dependent protection against glutamate toxicity compared to the untreated control. B355252 also displayed cell proliferative effect in the absence of glutamate (*p<0.05, ***p<0.001 compared to glutamate-treated cells; ###p<0.001, +++p<0.001 compared to control). Representative morphology of glutamate treated cells **(B)**, cells protected from glutamate toxicity by B355252 **(C)**, and untreated control cells **(D)**.

To assess the neuroprotective effect of B355252 under conditions of glutamate toxicity, HT-22 was challenged with 5 mM glutamate with and without pretreatment of B355252. The protective effect was analyzed with MTT assay 10 h after glutamate treatment. Cell viability in the glutamate treated population significantly declined by nearly 60% (p<0.001) compared to the untreated cells (Figure 2A). Pretreatment of cells with B355252 before glutamate exposure protected HT-22 from cell death by counteracting the toxic effect of glutamate. In the presence of 2 μM, 4 μM, and 8 μM compound, statistically significant increases in cell survival of 9.1% (p<0.01), 26.0% (p<0.001), and 61.9% (p<0.001) were observed respectively, compared to cells treated with glutamate only. Notably, at a concentration of 8 μM, B355252 fully protected HT-22 against the harmful effects of glutamate with cell viability attaining equivalent levels as that of the untreated control group. Interestingly, treatment with B355252 alone promoted cell proliferation by more than 16% (p<0.01) over control group.

We next confirmed these results in a qualitative visual fluorescent cell viability staining assay employing two fluorescent dyes, acridine orange (AO) and ethidium bromide (EB). AO/EB stains allow for rapid discrimination of viable and dead cells when visualized by fluorescence microscopy. AO is a cell-permeable dye that traverses the cell membrane and stains the nucleus of viable cells bright green, while EB stains dead cells red-to-orange, and is excluded by viable cells. Glutamate treated HT-22 cells evoked a significant decrease in cell viability as demonstrated by a sharp increase in the ratio of red to green cells observed in a phase contrast microscopy image (Figure 2B), confirming the data set obtained with the MTT assay. Conversely, pretreatment with B355252 protected HT-22 against glutamate injury as observed by the increase in the number of cells emitting green fluorescence (AO) relative to the red fluorescence of EB (Figure 2C). The AO fluorescence of B355252 protected cells closely matched the fluorescence of the control cells group (Figure 2D). These results show that B355252 has the functional capacity to rescue cells from glutamate evoked neurotoxicity, and possesses activity that promotes cellular proliferation.

### B355252 modulates GSH expression and depletes glutamate enhanced expression of AIF and Bax

**Figure 3:**
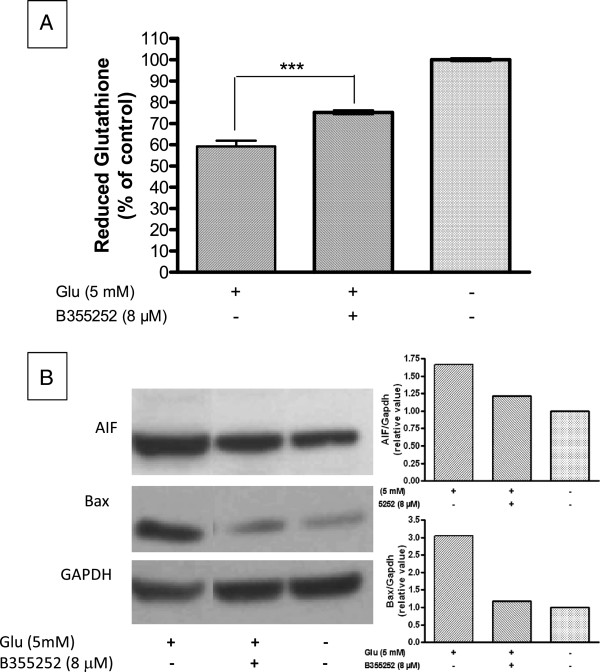
**B355252 partially restores glutamate-induced depletion of GSH and attenuates the increases in AIF and Bax expression in HT-22 cells.** HT-22 cells were pretreated with B355252 for 2 h prior to glutamate addition for 10 h as described in the Methods. The level of GSH was measured in the cellular lysates with monochlorobimane (MCB) and glutathione-S-transferase (GST). AIF and Bax protein levels were determined by immunoblot with anti AIF and anti Bax antibodies. GAPDH was used to normalize for variations in protein loading. **(A)**, Expression of GHS **(B)**, Immunoblots of AIF and Bax. The bars depict densitometric analyses of the relative values of AIF (top bar graph) and Bax (lower bar graph) present in glutamate treated, glutamate and B355252 treated, and control cells (***p<0.001 compared to glutamate-treated cells).

To ascertain how B355352 confers protection against glutamate-induced cell death we measured the level of reduced GSH, and two apoptosis associated proteins Bax and AIF in experimental and control cells, since glutamate has been documented to upregulate the levels of these proteins in neuronal cells. Glutamate treated cells showed a 40% (p<0.001) reduction of GSH in HT-22 compared to untreated cells (Figure 3A). Pretreatment of B355252 slightly reversed the effect of glutamate dependent-decrease of GSH in the cells by 15% (p<0.01) compared to glutamate treatment but was unable to restore GSH to comparable level as in untreated cells. In the case of the proapoptotic proteins AIF and Bax, the immunoblot analysis of cellular lysates revealed that glutamate significantly increased AIF in HT-22 cells by 40% compared to control cells (Figure 3B). This effect was reduced by 27% when cells were pretreated with B355252. Also, the expression of Bax by glutamate was significantly increased by more than 3-fold compared with expression in untreated cells. Pretreatment of B355252 before glutamate significantly blocked the expression of Bax protein, essentially reducing it to a level that is comparable to that of untreated cells. These results suggest that B355252 protects against glutamate-induced oxytosis and apoptotic cell death in HT-22 through a reversal of GSH depletion and robust repression of proapoptotic Bax expression.

### Exposure of cells to B355252 protects against glutamate-induced calcium overload

**Figure 4:**
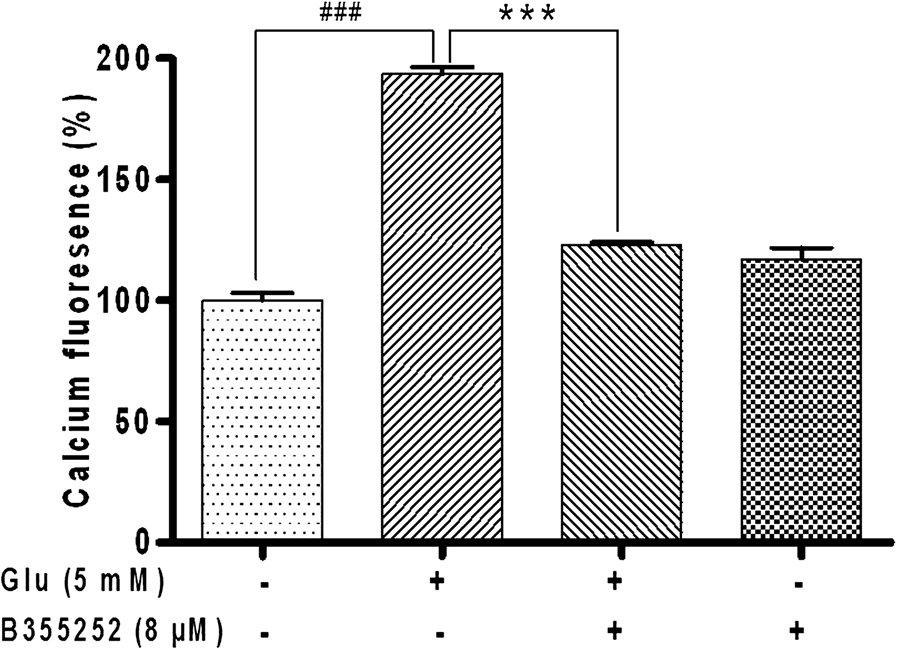
**B355252 inhibits glutamate-induced accumulation of intracellular Ca**^**2+**^**.** HT-22 was exposed to glutamate alone and in the presence of B355252. Changes in intracellular Ca^2+^ load was measured with the calcium binding dye Fura-2. Accumulation in Ca^2+^ by glutamate treatment was significantly attenuated by 8 μM of B355252. B355252 alone did not alter Ca^2+^ homeostasis in the cell. (***p<0.001 compared to glutamate-treated cells; ###p<0.001 compared to control).

In order to evaluate the effect of B355252 against excitotoxicity promoted by Ca^2+^ accumulation, HT-22 cells were treated with glutamate in the presence or absence of the test compound. The results indicate that glutamate significantly increased the level of intracellular Ca^2+^ as measured by the fluorescence of Ca^2+^-bound Fura 2-AM by 93% (p<0.001) compared to control cells (Figure 4). In the presence of B355252, the intracellular Ca^2+^ load declined significantly by nearly 71% (p<0.001) compared to cells treated solely with glutamate. This result implies that B355252 potentially prevents cell death and improves cell survival by modulating intracellular Ca^2+^ overload in HT-22 cells under glutamate insult.

### Glutamate-dependent accumulation of ROS is muted by B355252

**Figure 5:**
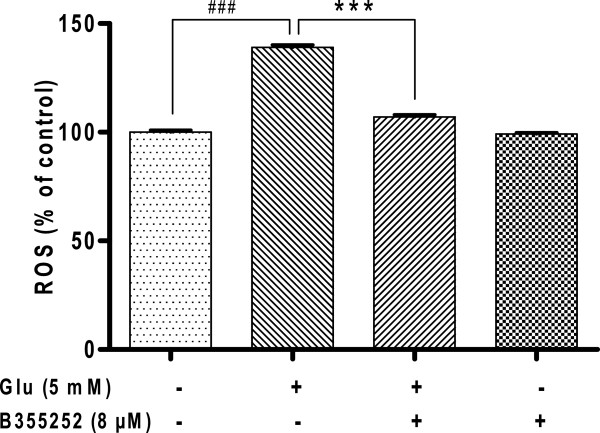
**Glutamate-evoked production of ROS is blocked by B355252 in HT-22 cells.** HT-22 was exposed to B355252 for 2 h, and then treated with glutamate for 10 h. ROS level was measured with CellRox Deep Red. Glutamate-dependent elevation of ROS was significantly attenuated by pretreatment of cells with B355252 (8 μM). B355252 alone did not significantly affect ROS level in the cell (***p<0.001 compared to glutamate-treatment; ###p<0.001 compared to control).

Since oxidative stress mediates glutamate induced cell death in HT-22 cells via ROS accumulation we examined the role of B355252 in oxidative stress promoted by glutamate toxicity. Intracellular ROS activity was measured after exposure of HT-22 to glutamate. As shown in Figure 5, the incubation of cells in glutamate stimulated an increase in intracellular ROS by nearly 40% (p<0.001) of control value. When the cells were pretreated with B355252 the level of ROS was significantly reduced by 40% (p<0.001) similar to the level observed in untreated cells, which suggest that B355252 protects cells against glutamate injury through the abatement of ROS production. Cells treated with B355252 alone exhibited no significant alterations in the burden of ROS.

### Erk phosphorylation is attenuated by B355252

**Figure 6:**
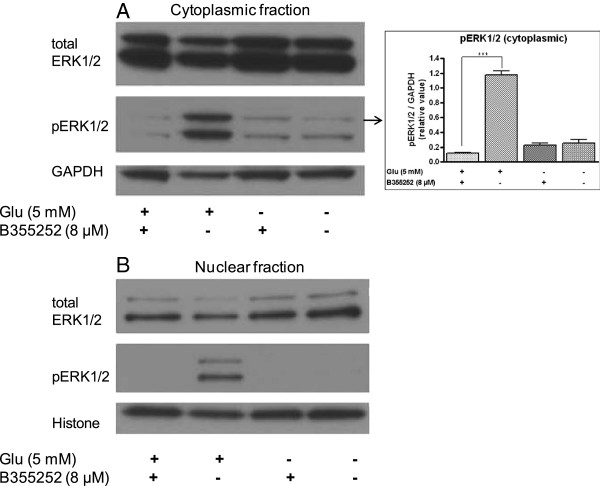
**Glutamate-dependent activation of Erk1/2 is blocked by B355252 in HT-22 cells*.*** Cultured cells were treated with glutamate 10 h following a 2 h pretreatment with B355252. Cellular fractions (cytosolic and nuclear) were prepared and subjected to immunoblot analysis with total Erk1/2 or pERK1/2 specific antibodies. Membranes were reprobed with GAPDH and H3 histone specific antibodies as controls for protein loading. **(A)**. Cytoplasmic fraction probed with total Erk1/2 (top), pERK1/2 (middle), and GAPDH (bottom) specific antibodies. Erk activation by glutamate was significantly attenuated by pretreatment of cells with B355252 (8 μM). B355252 alone had no effect on phospho-Erk1/2. The bar graph in figure **A** shows the relative level of active Erk in the samples. ***p<0.001 compared to glutamate-treated cells. **(B)** Nuclear fractions of samples probed with total Erk1/2 (top), pERK1/2 (middle), and H3 histone (bottom) specific antibodies. +/− indicates presence or absence of compounds.

Persistent activation of Erk1/2 by glutamate has been found to trigger cell death in primary cultures of rat cortical neurons and HT-22 cells [29-31]. To investigate whether prevention of glutamate toxicity by B355252 involves significant downregulation of Erk1/2 activity we measured the activation state of Erk1/2 by probing immunoblots of cytosolic and nuclear fractions of HT-22 with total and phospho-Erk1/2 (pERK) antibodies. Immunoblotting with anti-Erk1/2 in glutamate treated cells demonstrated that prolonged exposure of HT-22 to toxic levels of glutamate increased the total Erk1/2 proteins present in the cytosolic fraction by 12% of control (Figure 6A). However, the active form of Erk1/2 (pERK1/2) was drastically increased by 86% (p<0.001) in glutamate treated cells when compared to untreated cells. Similarly, there was no significant difference in the level of total Erk in the nuclear fraction of glutamate treated cells but a significant amount of pERK was present in the nucleus of glutamate treated cells (Figure 6B). When cells were pretreated with B355252, the immunoblots revealed no alterations in the expression levels of total Erk1/2 proteins in either the cytosolic or nuclear fractions compared to controls (Figure 6A and 6B). However, the glutamate evoked activation of Erk as observed with anti-pERK antibody was significantly reduced by 89.8% (p<0.001) in the cytoplasmic fraction upon treatment with B355252 and was not detected in the nucleus in comparison to glutamate exposed cells (Figure 6B). Exposure of cells solely to B355252 neither resulted in increased expression or activation of Erk1/2. These results confirm that glutamate alters the activation state of Erk1/2 in HT-22.

### Inhibition of Erk3 activation by glutamate is derepressed by B355252

**Figure 7:**
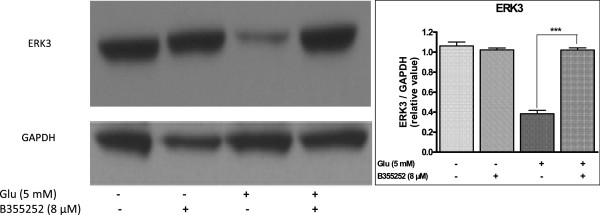
**B355252 restores glutamate-evoked reduction of ERK3 expression in HT-22 cells.** Cultured cells were treated with glutamate, with and without B355252. Cell lysates were prepared, subjected to SDS-PAGE, and immunoblotted with ERK3 specific antibody. Membrane was reprobed with anti-GAPDH and used to normalize gel loading. The bar graph shows the relative levels of Erk3 in the samples. Glutamate-dependent attenuation of ERK3 was reversed by treatment with B355252 (8 μM). B355252 exclusively had no significant effect on the basal level of ERK3 (***p<0.001 compared to glutamate-treated cells).

To further determine the influence of B355252 on other members of the Erk family that are highly expressed in neuronal cells, we analyzed the effect of B355252 on Erk3 in HT-22 cells under glutamate stress with immunoblot using anti ERK3. We tested the possibility that glutamate toxicity interferes with the expression of Erk3 and that B355252 could reverse the effect. Indeed when HT-22 cells were treated with glutamate the level of ERK3 was significantly decreased by 64% (p<0.001) compared to control cells (Figure 7). Pretreatment with B355252 altered the glutamate-dependent inhibition by restoring the expression of Erk3 to 96% (p<0.001) of the level observed in untreated sample. Sole treatment of HT-22 with B355252 did not significantly alter the cellular content of ERK3. This observation indicates that B355252 could indeed restore the level of Erk3 in neuronal cells under glutamate stress by directly blocking the effect of glutamate or upregulating the level of Erk3 in the cell.

## Discussion

Neurodegenerative diseases share a common mechanism of pathophysiology such as oxidative stress, mitochondrial aberrations, and inflammation, which lead to the degeneration and death of neurons. Developing therapeutics modulators of these universal mechanisms could have a significant impact in the management of these devastating diseases through delay of disease onset or disease progression. The interaction of glutamate with specific membrane receptors is responsible for many neurological actions mediated by neuronal cells in the CNS, including synaptic plasticity, sensation, and movement [32-34]. However, excessive glutamate can lead to neuronal cell death in a variety of pathological conditions which is thought to play a crucial role in the pathogenesis of many neuropsychiatric and neurodegenerative disorders [35]. In a previous study, we described the synthesis and identification of a novel phenoxy thiophene sulphonamide small molecule (B355252) enhancer of neurotrophin-dependent signaling, which promotes neurite outgrowth, extension, and cell survival [28], functions under assault in many neurological disorders. In the present study we demonstrate the ability B355252 to rescue HT-22 neuronal cells from glutamate-induced neurotoxic injury and sort to define the cellular events that underlie the rescue. Our results strongly suggest that B355252 prevents glutamate neurotoxicity through multiple effects targeted at mitochondria-dependent events including inhibition of Ca^2+^ overload, depletion of ROS, restoration of glutathione, and expression of the apoptotic proteins AIF and Bax. In addition B355252 exerted its effect by modulating the activity of typical and atypical the Erk family members.

Glutamate induces apoptosis at high concentrations in neurons, and HT-22 cells provide a model system to study glutamate-evoked death signaling pathways that enhance ROS formation and oxidative stress independent of NMDA-receptor.

These cells lack ionotropic glutamate receptors, but are still sensitive to high concentration of extracellular glutamate, which depletes glutathione and causes oxidative toxicity in an Erk-dependent manner. There is wide variation in the literature on the concentration of glutamate that induces oxidative toxicity in HT-22 cells. In these studies, the dose of glutamate used to induce cell death following 24 h treatment varied between 1 mM and 10 mM, while the rate of induction of cell death varied between 10% and 90% [30,31,36]. Based on the variations in glutamate concentration in these studies we determined the effective concentration of glutamate for our experiment in a dose response assay. Prolonged treatment with glutamate for 10 h triggered significant concentration-dependent cell death in HT-22 as measured by decrease in cell viability and EB fluorescence staining (Figure 1; Figure 2B). The B355252 dose-dependently protected the cells and consequently prevented the harmful effect of glutamate on HT-22 cells by restoring the cell health of HT-22 to comparable level as that of naïve cells at a concentration of 8 μM (Figure 2A). In addition to its neuroprotective attributes B355252 also exhibited intrinsic proliferative activity by stimulating cell growth in HT-22 neurons. These results indicate that cell death promoted by glutamate toxicity could be ameliorated by B355252 and support a neuroprotective role and therapeutic potential for the compound.

Substantial controversy exists in the literature with regard to how glutamate mediates its toxic effect in HT-22. Glutamate evoked oxidative death result in a time and concentration-dependent manner from mechanisms that involve both necrotic and apoptotic processes [30,37,38]. However, apoptosis appears to be more intimately involved in the process at late time points. Previous studies suggest that glutamate induces a particular type of a caspase-independent cell death called oxytosis, which involves the translocation of AIF from the mitochondria to the nucleus [39,40]. Immunoblot studies with anti AIF antibody show that AIF was marginally increased by glutamate treatment in the HT-22 cells (Figure 3B). This observation implies that AIF plays a role in glutamate-induced death but most likely does not represent a major cell death pathway in our current study, which is in agreement with previously published results [41]. The glutamate induced expression of AIF was inhibited to background level in the presence of B355252 (Figure 3B). It is unclear whether B355252 acts by direct perturbation of AIF translocation, at upstream regulators of AIF such as PARP, or whether the compound destabilizes released AIF and promotes its clearance in the cytosol.

Recent evidence has shown that exposure to glutamate regulates the expression of the proapoptotic Bax protein in HT-22 [41]. Bax specific inhibitors, antioxidants, and anti-inflammatory agents were capable of protecting against glutamate-induced cell death in neurons by blocking the expression of Bax [42,43]. In our study, immunoblots probed with Bax specific antibody show that glutamate stimulated increased expression of Bax in HT-22 (Figure 3B), which supports the conclusion that prolonged treatment of HT-22 cells with glutamate leads to apoptosis. This observation is in agreement with widely published data on the mechanism of cell death caused by glutamate exposure. Our results show that the glutamate-evoked Bax expression was sharply blunted by B355252. Based on the expression level in the presence of B355252, the substantial reduction in the amount of Bax to a large extent suggests that B355252 is a highly effective inhibitor of a major glutamate cell death pathway caused by the accumulation of proapoptotic Bax protein.

A major event during programmed cell death is an increase in cytosolic Ca^2+^[44]. Under normal physiological conditions glutamate-induced cell signaling intermediates such as Ca^2+^ influence a wide variety of cellular components and play a fundamental role in neuronal survival, differentiation, and development of synaptic circuits [11]. However, it has been shown that Ca^2+^ is a key mediator of numerous cell death pathways and that a complex relationship exists between mitochondrial function, ROS, Ca^2+^, and cell death [45]. Elevation of intracellular Ca^2+^ is a hallmark of excitotoxicity triggered by sustained or repeated glutamate exposure in neuronal cells. Ca^2+^ overload excessively activate Ca^2+^ signal transducers, which increase the vulnerability of neurons to cell damage or death. Previous studies have shown that inhibition of Ca^2+^ influx relieves glutamate neurotoxicity in HT-22 cells [46]. In the present study, intracellular Ca^2+^ in HT-22 cells was significantly elevated by glutamate treatment in agreement with past research findings. Subsequent treatment with B355252 caused a marked decrease in glutamate-induced Ca^2+^ overload (Figure 4). Since disregulation of the Ca^2+^ homeostasis has been identified as a key factor in glutamate toxicity [10,12], the observed effect of B355252 suggest that the compound interferes with glutamate activity or mechanistically restores Ca^2+^ balance in cells under glutamate assault leading to cell survival.

The production of ROS induced by oxidative stress has been noted in various studies of glutamate toxicity, suggesting that accumulation of ROS play a crucial role in the induction of cell death by glutamate. Previously studies show that protracted exposure of HT-22 to extracellular glutamate prevents cystine uptake into the cells via the cystine/glutamate antiporter, resulting in depletion of intracellular GSH. Both reduced GSH levels and increased ROS formation are established mechanisms that contribute to neuronal death in models of chronic and acute neurodegeneration. Reduced supply of glutathione, leads to influx of extracellular Ca^2+^ and accumulation of excessive amounts of ROS, which in turn leads to oxidative stress. Furthermore, elevated ROS level results in damage to macromolecules neurons. Excessive ROS must be promptly eliminated from the cell by a variety of antioxidant defense mechanisms that scavenge ROS if cells are to be protected from oxidative damage. In this study we observed that treatment of HT-22 with glutamate resulted in oxidative stress characterized by depletion of GSH, elevated production of ROS, and changes in cell morphology as reported in the literature. Pre-exposure of HT-22 cells to B355252 blocked glutamate-induced death through mechanisms that involve both increase in cellular GSH (Figure 3A) and reduction of ROS (Figure 5). Antioxidant scavengers such as N-acetylcysteine (NAC) and trolox prevent glutamate-induced cell death in HT-22 by sustaining cellular glutathione and reduction of ROS [30,47]. Thus, the present finding support the conclusion that B355252 acts as oxidant scavenger and the neuroprotection conferred on HT-22 may be dependent in part on its antioxidant attributes.

The superfamily of mitogen-activated protein kinases (MAPKs) which include extracellular signal-regulated kinases (Erks), c-Jun NH2-terminal kinase (JNK), and p38 MAP kinase modulate in a variety of cellular function in many cell types [48]. The Erk subfamily comprises 5 different isoforms, Erk1 to Erk5. Although Erks are traditionally viewed as a survival factor recent reports have demonstrated a death-promoting role for Erks in neuronal cells [30,31,49]. Erk1/2 has been implicated in glutamate-induced neuronal oxidative toxicity based upon the observation that U0126, a specific inhibitor of the Erk-activating kinase, MEK-1/2, protects both HT-22 cells and immature primary cortical neuron cultures from glutamate toxicity [31]. Administration of U0126 following focal ischemia in rodents led to a reduction in brain injury suggesting that Erk1/2 may also promote neuronal cell death as a consequence of acute injury *in vivo*[50]. Our results confirmed that U0126 could prevent glutamate-induced cell death in HT-22 by reduction of Erk phosphorylation (data not shown). Similarly, B355252 protected against glutamate toxicity via inhibition of Erk activation (Figure 6), but not JNK or P38 activation (data not shown), clearly demonstrating the involvement of Erk1/2 activation in the protection conferred by B355252. Furthermore, the activation of Erk1/2 in the glutamate excitotoxic model has been tightly linked to ROS production partly through Ca^2+^-sensitive signals [51]. These Ca^2+^-permeable pathways upregulates Ca^2+^ influx, which in turn activates several Ca^2+^-dependent kinases to increase Erk phosphorylation. However, some research reports have indicated that activation of Erk in HT-22 is independent of ROS accumulation. This conclusion is supported by the observation that U0126 was unable to block the generation of intracellular ROS during activation of Erk 1/2 in a glutamate excitotoxic model [31]. In the current study the production of intracellular ROS by glutamate and activation of Erk1/2 were significantly reduced in cells that are protected by B355252. These data support the view that B355252 unlike U0126 exerts it effects through multiple functional pathways, which influence glutamate-evoked activation of Erk1/2 and accumulation of ROS in promoting cell survival during glutamate toxicity. The mechanisms by which B355252 exerts these actions remain to be determined.

Erk3 is an atypical member of the mitogen-activated protein kinase (MAPK) family of serine/threonine kinases. Little is known about the biological function of Erk3 and even less about its regulation, substrate specificity, and cellular targets. Erk3 is abundantly expressed in neurons were it is found in both the cytoplasm and nucleus. Although its physiological functions remain to be established, signaling by Erk3 kinase has been theorized to play a role in neuronal morphogenesis and survival and in the regulation of cell growth and differentiation [52,53]. Recent work has shown that Erk3 interacts with and activates the MAP kinase-activated protein kinase MK5 and has been reported to inhibit S-phase transition in fibroblasts upon serum activation, which suggest that Erk3 may negatively regulate the cell-cycle depending on cellular conditions. However, it is unclear whether Erk3 regulates cell proliferation under physiological conditions. Research has shown that Erk3 kinase increases during differentiation of PC12 into neuronal lineage and that Erk3 mRNA is tightly regulated during mouse development, suggesting a role for Erk3 in embryogenesis [54,55]. Recently, Erk3 was found to form a ternary complex with MK5 and septin7 to promote dendrite development and spine formation in MK5 mouse knockout suggesting a role in the regulation of neuronal morphogenesis and survival [56]. In our study, glutamate treatment substantially blunted the expression of Erk3 in contrast to increased phosphorylation of Erk1/2. Treatment of cells with B355252 led to increase in the magnitude of Erk3, restoring the expression of the kinase (Figure 7). B355252 alone had no effect on the expression of pERK3, which suggests that pERK3 does not play a role in B355252-dependent cell proliferative activity. Taken together, the results of Erk regulation signify that B355252 protects HT-22 from glutamate-evoked neurotoxicity by opposing the deleterious effects of glutamate through coordinated restoration of typical and atypical Erk kinases.

## Conclusion

In this study we have shown that a novel phenoxy thiophene compound, B355252 protects against oxidative stress in a glutamate-evoked oxidative neurotoxicity model. Glutamate at toxic concentrations perturbs Ca^2+^ homeostatic mechanisms and leads to ROS generation. Also, excess glutamate depletes glutathione and upregulates the expression of AIF and Bax. In addition, glutamate targets the Erks, triggering opposing effects in the activation states of typical and atypical Erk kinases in HT-22 cells. The molecular mechanisms responsible for the protection of B355252 against glutamate injury in this neuronal cell line involves the restoration of Ca^2+^ homeostasis, suppression of ROS production, inhibition of AIF and Bax expression, and re-establishment of the dynamic interplay between the activation states of Erk1/2 and Erk3 kinases. In an earlier study we showed that B355252 possesses neuritogenic and NGF-dependent neurite outgrowth properties and our current finding demonstrate that this compound possesses robust antioxidant properties. Overall, the cumulative data on B355252 from our laboratory suggest that it is a promising small molecule with the potential for development as a therapeutic and neuroprotective agent for treatment of various neurodegenerative disorders.

## Methods

### Antibodies and reagents

Phospho-Erk1/2 (pERK1/2) rabbit monoclonal antibody, ERK3, and histone H3 rabbit antibody were purchased from Cell Signaling Technology (Danvers, MA) and Epitomics, Inc (Burlingame, CA). Goat anti-GAPDH [HRP] polyclonal antibody was from GenScript (Piscataway, NJ). L-glutamic acid monosodium hydrochloride was obtained from Sigma (St. Louis, MO) and B355252 was synthesized according to Williams et al. [28].

### Cell culture and treatment

HT-22 cells were maintained in Dulbecco’s modified Eagle’s medium (DMEM; ATCC, Manassas, VA) supplemented with 10% fetal bovine serum (FBS; Gibco BRL, Grand Island, NY), penicillin (100 U/mL), and streptomycin (100 μg/mL), at 37°C in a humidified atmosphere of 5% CO_2_. B355252 synthesized as previously described was prepared in DMSO at a stock solution concentration of 10 mM. The final concentration of DMSO was 0.1% in the cell cultures used in the present study. For experiments, actively growing cells were seeded at 2×10^4^ cells/well of 96-well culture plate or 5×10^5^ cells/well of 6-well cell culture plate and incubated for overnight prior to pretreatment for 1 h with compound and glutamate treatment for a period of 10 h.

### Assessment of cell viability

Cell viability was evaluated biochemically with the MTT (3-[4,5-dimethylthiazole-2-yl]2,5-diphenyl tetrazolium bromide) and visually with ethidium bromide (EB)/acridine orange (AO) fluorescent assay. The MTT assay is based on the capacity of cellular mitochondrial NADPH dehydrogenases to reduce the yellow water-soluble tetrazolium substrate into a dark blue/purple water-insoluble formazan product in viable cells. Cells seeded overnight in 96-wells plates were treated with glutamate with or without the B355252. At the end of the exposure period, MTT was added to a final concentration of 1 mg/ml to each well and the plates returned to the incubator for 3 h. The medium was carefully removed, the cells rinsed once with PBS and 150 μL DMSO was added to lyse the cells. Components of the wells were mixed thoroughly with repeated pipeting until the formazan crystals were completely dissolved. Changes in the absorbance of formazan dye in live cells were measured using a PheraStar multipurpose plate reader (BMG Labtech, Durham, NC) at 490 nm. The extent of MTT conversion in treated cells was expressed as a percentage of the viability of the control cells.

For the fluorescent visualization assay, EB/AO solution was prepared as a 100X stock solution containing 1 mg/mLEB and 0.3 mg/mL AO in 2% ethanol and stored at −20°C in 1 mL aliquots. HT-22 cells were cultured in 6-well plates and treated as described above. At the end of the incubation period, the samples were rinsed with PBS, stained with a cocktail of AO/EB diluted to 1X in phenol free DMEM, and immediately document by fluorescence microscopy utilizing green filter for AO (488/520 nm; ex/em) and red filter for EB (510/595 nm; ex/em).

### Measurement of reduced glutathione

GSH content was assayed with the monochlorobimane (MCB) glutathione detection kit (Biotium, Hayward, CA). MCB is non-fluorescent dye in with high affinity for GSH. MCB becomes highly fluorescent (ex/em=390/490) upon reacting with GSH in the presence of glutathione-S-transferase (GST). To assay for GSH content, cells cultures treated with B355252 and/or glutamate as previously described were detached and centrifuged in a microcentrifuge tube at 700 × g for 5 min. The cells were washed once with ice cold PBS at 4°C and assayed according to the protocol provided by the kit manufacturer. Reduced glutathione was used as a positive control.

### Measurement of intracellular Ca^2+^ increase

HT-22 cells were cultured and treated as described for the viability assessment assay. After incubation with B35525, control and drug-treated cells were washed with PBS and loaded with 5 μM Fura-2AM for 45 min at 37°C. Loaded cells were washed twice with DPBS and the amount of intracellular Ca^2+^ was determined in a SpectraMax Plus384 (Molecular Devices) by successive excitation of the Fura-2 dye with a xenon light source at 340 and 380 nm. The emitted fluorescence was passed through a 510-nm filter, recorded and analyzed with SoftMax Pro software. The concentration of intracellular Ca^2+^ was calculated by averaging the ratio of fluorescent signal acquired at 340 and 380 nm and expressed relative to values of control wells.

### Measurement of reactive oxygen species in live cells

ROS, the cellular marker of oxidative stress was detected using the cell permeable fluorogenic probe CellROX Deep Red (Invitrogen, Carlsbad, CA) that emits red fluorescence upon oxidation in cells treated with glutamate with and without B355252. Incubation of the cells with B355252 and glutamate was performed as described for previous assays. The amount of intracellular ROS was determined by incubating cells with 5 μM CellROX reagent for 30 min at 37°C. The media was removed and the cells washed twice with DPBS. ROS level was measured with the PheraStar (BMG Labtech., Durham, NC) at 640/655 nm, the excitation/emission maxima for CellRox and expressed as a percentage of control.

### Immunoblot analysis

Sub-cellular fractions (cytosol and nuclear) were extracted from treated and control cells by resuspension of cells for 5 min in ice cold cell lysis buffer containing 20 mM Tris pH7.4, 10 mM KCL, 3 mM MgCl2, 0.5% NP40 and protease inhibitor cocktail (EMD Millipore, Billerica, MA). The cells were lysed by repeated mixing on ice with pipet. The lysates were transferred to microcentrifuge tubes and centrifuged at 2,000 × g for 10 min. The resulting supernatant was stored as the cytosolic fraction. The pellets were washed twice in cell lysis buffer, resuspended in nuclear extraction buffer (cell lysis buffer supplemented with 1% SDS), sonicated briefly on ice and centrifuged at 20,800 × g for 30 min at 4°C. The supernatants were saved in clean ice cold tubes as nuclear fractions.

Protein concentrations were determined with the Bradford reagent and 20 μG of protein per sample was loaded on 10% NuPAGE BT gels (Invitrogen), subjected to electrophoresis, and transferred to a PVDF membrane (EMD Millipore). The blots were probed with monoclonal antibodies to pERK1/2 and ERK3, and incubated with enhanced chemiluminescent (ECL) goat anti-rabbit IgG conjugated to horse radish peroxidase (HRP) as secondary antibody. The antigen/antibody complexes were detected with SuperSignal West Pico Chemiluminescent Substrate (Thermo Scientific, Rockford, IL) and exposed to X-ray Films. To control for gel loading, membranes were probed with anti GAPDH (cytosolic) or anti Histone H3 (nuclear) antibodies.

### Statistical analyses of data

The data are expressed as percent of mean values ± standard deviation (SD) relative to the controls from at least 3 independent experiments (n=6). Statistical analysis of results was performed in GraphPad PRISM (GraphPad Software Inc). For experiments involving more than two groups, statistical evaluation of the data was performed using one-way ANOVA followed by Bonferroni post test analysis. A value of *p* <0.05 was considered to be statistically significant.

## Competing interests

The authors declare that they have no competing interests financial or otherwise.

## Authors’ contributions

GCI conceived, supervised, and prepared this manuscript describing the finding of the study. NSG and EYH conducted the experiments in this study. All authors read and approved the final manuscript.
